# Implementation facilitation of the “11 for Health in Denmark”: A case study in a Danish 5^th^ grade class

**DOI:** 10.1111/sms.14069

**Published:** 2021-10-10

**Authors:** Esben Elholm Madsen, Peter Krustrup, Trine Kjeldgaard Møller, Tina Hansen, Malte Nejst Larsen, Mads Madsen, Henrik Kruse Hansen, Anne‐Marie Elbe, Carsten Hvid Larsen

**Affiliations:** ^1^ Department of Sports Science and Clinical Biomechanics University of Southern Denmark Odense Denmark; ^2^ Department of Midwifery, Physiotherapy, Occupational Therapy and Psychomotor Therapy University College Copenhagen Copenhagen Denmark; ^3^ Faculty of Sport Science Institute of Sport Psychology and Physical Education Leipzig University Leipzig Germany; ^4^ Danish Institute for Advanced Study (DIAS) University of Southern Denmark Odense Denmark; ^5^ Physical Medicine and Rehabilitation Research – Copenhagen Department of Physiotherapy & Occupational Therapy Amager‐Hvidovre Hospital Hvidovre Denmark

**Keywords:** 11 for Health in Denmark, case study, children, football, physical activity, school setting

## Abstract

The “11 for Health in Denmark” concept aims to enhance 10–12‐year‐old schoolchildren's physical activity levels and health knowledge through an 11‐week football intervention and has been shown to induce positive effects in numerous areas. However, little is known about the implementation facilitation of this concept in schools. This case study therefore aims to gain a deeper understanding of the implementation facilitation of “11 for Health” in a Danish 5^th^ grade class comprising 22 schoolchildren (12 boys and 10 girls, *M*_age_ 11.86±) and one teacher. Data were derived from multiple school situations using photographs and videos, observations, and informal interviews. Five themes were identified: (1) environmental and organizational factors impacting on the implementation; (2) familiarization with the concept for the teacher and schoolchildren; (3) previous clique formation; (4) the central role of the teacher; and (5) the importance of how praise partners are allocated. We found high adherence to the “11 for Health” concept, in which the teacher played a key role by applying an autonomy‐supportive style of teaching and using the praise partner concept in a suitable manner. A clique of football‐playing boys was a resource, as their praising of classmates encouraged adherence. However, familiarization with the “11 for Health” concept was a challenge and the clique's competitive nature occasionally built barriers to other schoolchildren without football experience, potentially leading to a more controlled form of motivation. Based on the results, we encourage teachers to apply autonomy‐supportive teaching when applying the “11 for Health” concept.

## 1 INTRODUCTION

The World Health Organization (WHO) recommends that children and adolescents should engage in a minimum average of 60 min/day of moderate‐to‐vigorous intensity, mostly aerobic, physical activity (PA) across the week.^1^ However, only 26% of Danish children between the ages of 11 and 15 conform to national guidelines of 60 min of moderate to high daily PA levels.^2^ During school hours, PA levels and sedentary behavior vary greatly, and for 5^th^ grade schoolchildren in the least active schools only 37.3 min or less is spent on PA during an average school day.^3^ There are many ways to increase children's daily PA,^4^ and throughout the years, a number of large school‐based interventions have been conducted to increase PA. For example, one school‐based program found that expanding physical education (PE) lessons could improve physical capacity,^5^ while a school‐based multi‐component PA intervention elevated PA and fitness, and reduced adiposity.^6^ As examples from a Danish context the Svendborgproject—a program that proved to triple and improve the amount of PE was successfully implemented and sustained by the Danish municipality of Svendborg over a period of 10 years (2008–2018) to improve PA,^7^ while the Move for Well‐being in Schools (MWS) developed, implemented and evaluated a school‐based PA intervention for 4^th^ to 6^th^ grade schoolchildren.^8^ The key factors behind the successful implementation and sustainability of the Svendborgproject were identified as adapting to existing school practice, clear requirements, not threaten the upkeep of academic standards, regular status meetings, and training courses when analyzing the provider and intervention characteristics of the project.^7^ Within the MWS project several factors including having a strong focus on implementation, knowledge translation mechanisms, and ongoing dialog with the schools were found to affect the implementation.^9^ As a way of responding to the ongoing challenges of low PA, in 2006 the Fédération Internationale de Football Association (FIFA) and FIFA’s Medical Assessment and Research Centre (F‐MARC) developed a concept for children entitled “FIFA 11 for Health”.

In 2016, an adapted “FIFA 11 for Health” program was implemented in Danish schools named “11 for Health in Denmark” (hereafter “11 for Health”).^10^ Since then, several studies targeting 5^th^ grade schoolchildren have reported positive effects on the participants’ physical fitness, cognitive performance, well‐being, enjoyment, and health knowledge.^10^, ^11^, ^12^, ^13^ Specifically, Lind et al.^12^ found that the “11 for Health” concept significantly improved psychomotor function and attention. Madsen et al.^14^ found a significant increase in physical well‐being. Larsen et al.^11^ found the “11 for Health” concept successful at increasing health knowledge and enjoyment, with only around 5% of the schoolchildren not liking the concept. In conjunction with the “11 for Health” concept, Ørntoft et al.^15^ found that schoolchildren engaged in club‐based ball‐game activities had higher exercise capacity, lower resting heart rate, and higher muscle mass than schoolchildren not engaged in leisure‐time sports. However, all of the above‐mentioned studies adopted a quantitative approach focusing on the effects of the “11 for Health” concept on measurable variables in schoolchildren. Consequently, a deeper understanding is needed of what actually happens during the implementation facilitation of the concept and of the experiences of teachers conducting the intervention. Closing this knowledge gap could help healthcare authorities, schools, and teachers by providing further insight into the challenges that might arise during implementation and how it can be ensured that the intended positive effects of the “11 for Health” concept are actually achieved.

### 1.1 The “11 for Health” concept

The “11 for Health” concept is designed for 10–12‐year‐old 5^th^ grade children and can be described as a health education program that takes place on the football pitch. The concept combines health education and PA designed as small‐sided games or technical drills in small groups (eg, visualizing healthy habits by dribbling a ball without hitting cones that represent cigarettes). As shown in Table 1, the concept consists of two weekly 45‐min sessions over an 11‐week period and has recently been implemented in multiple schools in Denmark. The teacher implementing the “11 for Health” concept normally decides which school lessons the two sessions should replace, with one of the sessions often replacing PE, and the other replacing another subject (eg, maths or science). Each week the training focuses on delivering one of ten health messages, ending with a final round‐up week (week 11). The sessions aim at an intense level of PA and include team exercises and group discussions on health topics. A key element of each session is the concept of praise partners. Each week the schoolchildren get a new praise partner, and at the end of each session, praise partners briefly get together to praise each other on their accomplishments during the session.^10^ Before teachers are able to teach the “11 for Health” concept, they are required to participate in a training course hosted by researchers from the University of Southern Denmark (SDU) in collaboration with the Danish Football Association (DBU). The training course lasts 2 days and its purpose is to ensure that the teachers deliver the “11 for Health” concept in an engaging and age‐, gender‐ and culture‐sensitive format.^12^

**TABLE 1:** “11 for Health in Denmark” program: session activities, health messages, and topics^10^

Week	“Play football” activity	“Play fair” activity	Session topics
1	Warming up	Play football	Prepare for exercise and sport
2	Passing	Respect others	Respect and help others and avoid bullying
3	Goalkeeping	Be active	Walk, cycle, use the stairs in daily life
4	Dribbling	Avoid drugs, alcohol, and tobacco	Avoid unhealthy addictions
5	Controlling the ball	Control your weight	Control the quantity of food eaten
6	Defending	Wash your hands	Develop good hygiene
7	Trapping	Drink water	Drink water instead of soft drinks
8	Fitness training	Eat a balanced diet	Train and eat a varied diet
9	Overlapping	Keep fit	Do vigorous exercise
10	Shooting	Think positively	Have a positive mindset
11	Teamwork	Fair play	Review all health issues

### 1.2 The educational development environment

**FIGURE 1:**
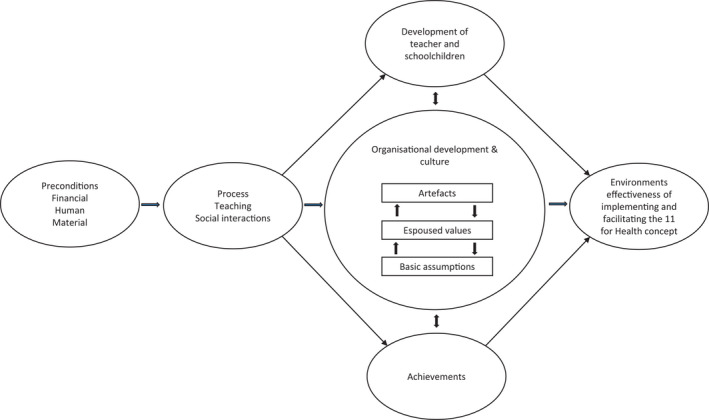
Explanatory working model adapted from Henriksen, Stambulova, & Roessler^17^

To gain a deeper understanding of the implementation facilitation of the “11 for Health” concept a description of the school environment is needed. We therefore chose to employ an ecological description focusing exclusively on the school domain and teaching. According to Henriksen, Stambulova and Roessler,^16^ such environments can be defined as a dynamic system comprising: (a) a schoolchild's surroundings at the microlevel where academic and personal development take place, (b) the interrelations between these surroundings; (c) the macro‐level, ie, the larger context in which surroundings are embedded; and (d) the organizational culture of the school and class, which is an integrative factor of the environment's effectiveness for facilitating implementation of the “11 for Health” concept. Inspired by Henriksen, Stambulova and Roessler,^16^ our framework consisted of two models. The first model—the descriptive working model—comprised the development environment of the schoolchildren, which we used to describe and clarify the tendencies, roles and functions of the various components and relations within the school domain. The second model—the explanatory working model adapted from Henriksen, Stambulova and Roessler,^17^ outlined in Figure 1—comprised the factors within the environment, which can be described as specific structured factors affecting the implementation facilitation of “11 for Health” and potentially providing the environment success. The explanatory model illustrates how the daily routines and processes (eg, PE sessions and classes) have three interrelated outcomes: the development of the teacher and schoolchildren, achievements, and organizational development and culture. “Preconditions” include financial factors, human factors (eg, resources in the form of teachers and schoolchildren), and material factors (eg, training and school facilities). “Process” refers to everyday activities in the school environment. “Development of teacher and schoolchildren” refers to the acquisition of skills present and/or derived from “11 for Health”. “Achievements” refers to the success of the implementation facilitation of the “11 for Health” concept. “Development of teacher and schoolchildren” can be seen as a product of organizational status, development, and culture of the implementation facilitation of the “11 for Health” concept. According to Schein,^18^ “Organisational development and culture” consists of three levels: cultural artifacts, espoused values, and basic assumptions. “Cultural artifacts” include stories, myths, customs, and traditions communicated within the environment, and also physical cultural manifestations such as buildings and surroundings. “Espoused values” can be seen as social principles, class customs, norms, goals, and standards that the environment shows. “Basic assumptions” are the teachers’ and schoolchildren's underlying reasons for actions, and consist of taken‐for‐granted beliefs and assumptions. The working model predicts that the implementation facilitation of the “11 for Health” concept can be seen as a result of the interplay between preconditions, process, individual and class development, and achievements with cultures serving to integrate these different elements.

Accordingly, this study aimed to gain a deeper understanding of the implementation facilitation of the “11 for Health” concept in a Danish 5^th^ grade school class. The objectives of the study were (a) to provide an ecological description of the school environment and (b) to examine and identify factors in the implementation and facilitation of the concept.

## 2 MATERIALS AND METHODS

The study was designed as a case study to holistically capture the complexity of a single‐bounded case.^19^ The present study is seen as *instrumental* because it provides a comprehensive understanding of the factors involved in implementing and facilitating the “11 for Health” concept,^20^ and it is *intrinsic* ^21^ because it gives a structural understanding of this particular case. Thus, we do not engage in value‐free assumptions or neutral observations, realizing the influence of the researchers on the research. Instead, we acknowledge that reality is conceptually dependent meaning that it can be falsely characterized and falsely categorized.^22^ The importance of being self‐reflective to increase integrity and rigor is important.^23^ Thus, aligned with a critical‐realist epistemological standpoint, and due to our extended knowledge of the “11 for Health” concept, we believe it was not feasible to stay objective and neutral in our interpretations of the data collected as the domain of the real cannot be reduced to the domain of the actual.^22^ Therefore, the background of the authors will be described to inform the reader how interpretative insights might be influenced. The majority of the authors primarily conduct football research and have experience of football at various levels. These backgrounds are somewhat reflected in the “11 for Health” training manual and the teaching during the course, which is described and taught using “football language” (eg, “make an overlap,” meaning when a player runs up from behind another player, goes past them and then receives the ball). However, the experience of the researchers behind the “11 for Health” concept of how the concept was implemented and facilitated was, at the time of the study, limited.

### 2.1 Procedure and case selection

Prior to the main study, the first author conducted a 6‐week pilot study following the teaching of the “11 for Health” concept at another school through the autumn of 2017. The main criteria for selecting this specific pilot case were the school's accessibility (closely located geographically), prior teacher contact, and an openness about facilitating implementation of the “11 for Health” concept. The pilot data provided insights into the basic issues being studied, which led to a realization of the need to be flexible in different areas but also raised awareness on sharpness and on reflection and documentation during and after the observational work.^24^

After the pilot study, another school was chosen based on the information‐oriented case selection method known as paradigmatic case selection, which highlighted more general characteristics of the school in question.^25^ Based on our prior knowledge about schools participating in the “11 for Health” concept, this case was identified as having somewhat prototypical values. These values were identified as being located in a typical Danish suburb, having children with an inhomogeneous socioeconomic background, and having a teacher without any prior football knowledge and coaching experience. The observation was carried out over a 4‐month period from March to June 2018, usually over the entire school day (from 8 am to 3 pm) and on 2 days a week. Days with “11 for Health” teaching were targeted.

### 2.2 Participants

The participants were 22 Danish 5^th^ grade schoolchildren and one female teacher from the Capital Region of Copenhagen, Denmark (12 boys and 10 girls, *M*_age_ 11.86±). The teacher voluntarily signed up and took part in the “11 for Health” teaching course in January 2018.

### 2.3 Research methods and instruments

The unit of analysis when seeking to gain a deeper understanding of the implementation facilitation of the “11 for Health” concept in a Danish 5^th^ grade school class can be seen as the interplay between preconditions, process, individual and class development, and achievements. To achieve this aim, a case study was utilized, which can be defined as an empirical inquiry that investigates a real‐life phenomenon within its real‐life context^26^ by using different sources of data that can be used as proof of evidence.^27^ To increase validity, as the strengths of one approach can compensate for the weaknesses of another,^28^ a triangulation of data sources was used, including digital photographs and filming, observations, and short informal interviews.

#### 2.3.1 Digital photographs and filming

Using a combination of digital photographs and filming enables researchers to understand not just what the participants say they do, but also what they actually do, in everyday life.^29^ As filming might be less intrusive than a note‐taking evaluator^30^ and provide significant insights into what participants are focusing on, thinking about, or how they are feeling (eg, overt body language and self‐talk)^31^ the authors decided to film and photograph observations at the football field using a cell phone. After each day of observations, data were reviewed, described, and transcribed linguistically to the computer. After transferring the data to a secure university server in line with the APA ethical principles,^32^ all material was deleted from the cell phone. In total, 108 photographs were collected and around 3 h of videos were filmed. The videos were distributed on 87 video clips with an average duration of 2 min 1 s.

#### 2.3.2 Observation

The aim of the observation is to provide information about the topic being studied. The pilot study revealed that the observational role when following the teaching of the “11 for Health” concept alternated on a continuum between participating observer, observer participating, and occasionally, observing teacher.^30^ The participant observations were conducted in the school courtyard, on a grass field near a harbor and on a grass field near a tennis club. The formal observation was conducted in the classroom and in the hallway in front of the classroom.

#### 2.3.3 Informal interviews

The interviews can be described as brief discussions or casual conversations to provide further insights when an opportunity arises. In some cases, they consisted of single question to get a sense of how activities were experienced. At other times, longer questions were used to introduce discussions at the end of an observation period.^20^

### 2.4 Data analysis

When selecting a general analytical strategy for the case study, thematic analysis was used to jointly identify, analyze, and report patterns (themes) within the multiple data sources (ie, observations, videos, and short informal interviews). The thematic analysis involved searching across a data set to find repeated patterns of meaning.^33^ The themes were tentatively identified in an inductive or “bottom‐up” way. This meant that the themes were data‐driven, meaning a latent level of identifying themes.^33^ As shown in Table 2 ^,^ the analytic process followed the six phases proposed by Braun and Clarke,^33^ with the notion of contemplating the process as being recursive, where we moved back and forth as needed throughout the phases.

**TABLE 2:** Phases of the thematic analysis

Phase	Description of the process
1. Familiarizing with the data:	Transcribing data and watching videos, reading/watching and rereading/rewatching the data, looking for potential meaning, and issues of potential interest.
2. Generating initial codes:	Initial coding identifying features of the data (text and videos) that appeared interesting in relation to the research question. As an example of data extract: “Simone and Mohammed are holding hands and running after the ball in the school courtyard. Simone: “Stop stop Mohammed, I can't keep up with you”. Simone laughs out loud, and Mohammed stops and starts to laugh too”.
	This data extract was initially coded as smiles and laughs which later became a sub‐theme.
3. Searching for themes:	Collating and sorting codes (text and videos) into potential themes using a mind map in Nvivo ending with a collection of candidate themes and subthemes.
4. Considering and reviewing themes	Reading and considering themes and subthemes in terms of adequately forming a coherent pattern by creating an initial thematic map. This process was repeated using the entire data set
5. Defining and redefining themes	Analyzing, defining, and refining the themes in accordance with the research question and the overall story of the analysis. Clear names and definitions were generated.
6. Producing the fully worked‐out themes for the result section	Final names and definitions were given for 5 themes, including compelling extracts examples and analysis relating back to research questions.

The first phase started during the data collection, when the first author looked for and noted patterns of meaning and issues of potential interest. To maintain an inductive approach, the reading of relevant analytic literature began in follow‐on from the data collection. During and after the data transcription, the first author repeatedly looked at the videos and field notes and began taking notes for future coding. After familiarization with all aspects of the data, the linguistic data were transferred to NVivo^34^ ^,^ and the schoolchildren's names were pseudonymized. Data were organized by time, beginning with the first day of observation. The first author coded the transcription in a manner accounting for all verbal and some nonverbal utterances, and situational actions, such as exercises on the football field and incidents in the classroom, were described thoroughly. In the second phase, the initial codes from the data extracted to NVivo^34^ were produced. These codes identified a feature of the data that appeared interesting to the authors and referred to the most basic segment of the raw data that could be assessed in a meaningful way regarding the phenomenon. The authors kept as many potential themes/patterns as possible, as it was not known at this point what would be interesting later on. In phase three, the authors collated and sorted the coding list into potential themes using a mind map. This phase produced a collection of candidate themes and sub‐themes, with all extracts of data that had been coded about them. In phase four, the collated extracts for each theme and subtheme were read and considered in terms of appearing to form a coherent pattern adequately capturing the contours of the coded data.^33^ The authors did this by creating an initial thematic map. Second, to consider whether the initial thematic map accurately reflected the meanings evident in the data, the authors repeated the same process using the entire data set. In phase five, themes were analyzed, defined, and refined following the essence of what each theme was about by identifying what was interesting and why in relation to the aim of the study, the ecological model and the overall analytical story to ensure there was not too much overlap. In this phase, clear names and definitions were generated and the authors identified whether a theme contained any subthemes. Phase six began with fully worked‐out themes telling a story of the data in which each theme was embedded within an analytic narrative that compellingly illustrated the story about the data.

### 2.5 Ethical approval

The study was approved by the Regional Committees on Health Research Ethics for Copenhagen and Southern Denmark (J.no H‐16026885). Prior to the study, the schoolchildren's parents attended an informational meeting^10^ in which they were specifically informed about the research methods and instruments being used (eg, using digital photographs and filming), and written parental consent was obtained. Furthermore, prior to the data collection, the schoolchildren provided their consent when being informed about these procedures in the classroom.

## 3 RESULTS

Overall, the implementation facilitation showed high adherence to the “11 for Health” concept, as all lessons described in Table 3 took place and all schoolchildren participated and rarely missed class. The main reasons for the high adherence were the motivational style of teaching and the fact that most of the schoolchildren seemed to enjoy the “11 for Health” concept. Nevertheless, there were challenges during the implementation. One challenge was the need to familiarize the children with the games and drills within the concept, which led to frustrations from some of those boys who were active football players at the beginning of the 11 weeks of teaching. Also, a previously existing clique of football‐playing boys who occasionally focused merely on results rather than on success and enjoyment sometimes was experienced as challenging for the teacher and the other schoolchildren. This competitiveness was at times found to build barriers to other schoolchildren without football experience, potentially leading to a more controlled form of motivation.

**TABLE 3:** Coding framework: a deeper understanding of the implementation facilitation of the “11 for Health” concept in a Danish 5^th^ grade class

Candidate themes	Sub‐themes
1) Environmental and organizational factors impacting on the implementation	Organizational and physical level
	No football pitch
	Support from headmaster and teacher colleagues
	The concept was taught wherever possible
2) Familiarization with the concept for the teacher and schoolchildren	Frustration
	Confusion
	Difficulty
	Anger
	Increasing familiarity
	Providing feedback
3) Previous clique formation	Taking account of each other
	Familiarity with football language
	Positive and negative ways of using a football language
	Competitiveness
	Barriers
	Dissatisfaction
	Feeling reluctant
4) The central role of the teacher	Stimulate enjoyment and interest
	Schoolchildren smiling, laughing, showing excitement, and giving high‐five
	Praise partners
	Behaving enthusiastically
	Some schoolchildren “just” behaving in line with the expectation
5) The importance of how praise partners are allocated	Using creative and neutral ways to assign praise partners
	Portraying schoolchildren in a different light
	Finding new social constellations
	Praising gradually become integrated within the class
	Supporting competence and relatedness

### 3.1 Environmental and organizational factors impacting the implementation

The first theme we identified referred to the macrolevel in the ecological model and the environmental and organizational impact. At this level, we found that the school environment had a profound impact on the implementation facilitation of “11 for Health”, as the concept required the teacher's head teacher and colleagues to be flexible in adjusting classes and subjects to fit the “11 for Health” concept into the school curriculum. The “11 for Health” teacher received structural support from the head teacher and her colleagues, who endorsed the implementation facilitation of the “11 for health” concept. However, we found it was an ongoing challenge to fit the “11 for Health” concept into a strict school timetable, which allowed little flexibility to take time from regular subjects such as maths or science. At a physical level, we found the school did not have access to a regular football pitch, which meant the teacher was obliged to use other possible areas. This meant that the concept was taught, wherever possible, in the areas surrounding the school, such as in the school courtyard, on a grass field near a harbor and on a grass field near a tennis club. These alternative locations were also used for outdoor informational lessons related to the “play fair” sessions. The school courtyard had trees and small areas of grass, but because a lot of people used the courtyard, it could best be described as a big dirt area in which the grass was worn away, making it far from ideal for football. Nevertheless, we found that the schoolchildren still enjoyed the “11 for Health” concept, despite being taught in all sorts of locations, having to retrieve the ball from beneath bushes, or being stung by nettles.

### 3.2 Familiarization with the concept for the teacher and schoolchildren

In respect of the microlevel of the ecological model, the implementation facilitation of the “11 for Health” concept required preparation and familiarity before the teacher and the schoolchildren could utilize the concept. It took quite a few weeks and repetitions before they became familiar with the games and drills, and the teacher was able to provide a rationale for the activities. Our analysis revealed that this period was especially challenging for some of the boys with previous football experience. At times, these boys found it frustrating committing to and accepting an unknown game format, which was experienced differently from playing football with two goals in a regular football club setting and/or playing with friends during leisure time. This became clear to us in the first week of teaching, when some boys autonomously chose to combine the instructions from the “11 for Health” concept aimed at a high level of PA and continuing the game when scoring while also utilizing a football‐specific behavior that involved keeping score and winning. For one of the boys, Willie, these impulsive choices of some classmates led to feelings of confusion and frustration, and in the end to an outburst of anger observed by his sudden willingness to tackle an opponent aggressively. However, such challenges were often overcome by the teacher, as she was able to interact and provide feedback drawing on her increasing familiarity with the concept. This familiarity, derived from the teaching course and previous teaching and game experiences, gradually made her competent and capable of referring to and explaining how games and drills were supposed to be performed.

### 3.3 Previous clique formation

In respect of the micro‐level of the ecological model, another theme refers to a previous clique formation affecting the implementation facilitation of the “11 for Health” concept. We found that a group of seven boys had formed a clique, uniting around an autonomous eagerness to rganizes and play football whenever possible. For these boys, football had personal meaning and involved an emotional commitment, and they were experiencing a sense of autonomy, seemingly with a reduced sense that the teacher was controlling their behavior. The impact from the espoused values from the football clique, and football in general, was also visible elsewhere, as we found a familiarity among all schoolchildren with a football‐specific communication culture using certain verbal and bodily football language. This language was used both positively and negatively, sometimes at the same time. When used positively, it mostly revolved around spontaneous praising in different ways and showed that the clique was supporting a relatedness around football, as they often took account of other schoolchildren who were not part of the clique. We found examples of the football clique adjusting passes, shots, and tackles based on an awareness of whether other children were competent at football or not. However, the football language was also used negatively, and we also found that members of the clique played hard and rough against other boys within the clique and were highly competitive, focusing on picking the best possible team to win. Occasionally these behaviors built barriers to schoolchildren outside the clique, with some children labelled as having inferior football skills. An example of this was observed in a situation involving four children named Simone, Bernard, Sean and Morris. In this situation, Simone remained unpicked for a team and Bernard, Sean and Morris, members of the clique, were discussing which team should pick her:

Bernard: “Morris and Sean, you have to pick Simone for your team!”

Morris: “No, we won't.”

This type of behavior from the three boys sometimes made children outside the clique feel reluctant about the game. Particularly, hectic football situations led to more negative experiences, as the football language at times became clearly confrontational and unpleasant. Another situation was observed where a boy named Mohammed shouted and looked angrily at a girl named Sallie during a game: *“If we don't pass the ball to each another*, *we're not going to be able to score!”* Sallie was not able to understand the meaning of Mohammed's instructions and felt somewhat shocked by his harsh line of language. She reacted by withdrawing on the field for some minutes, although she was eventually encouraged by other classmates to resume the game.

### 3.4 The central role of the teacher

Another theme also relating to the microlevel of the ecological model was the central role of the teacher. The teacher was found to demonstrate a motivational style of teaching, accompanied by understanding and support, which stimulated the schoolchildren's experience of enjoyment and interest both within the class and during the “11 for Health” concept. This behavior was highly visible during the “11 for Health” sessions and often resulted in the children smiling, laughing, showing excitement, and giving high‐fives. Examples of this were observed when the teacher enthusiastically encouraged the children to support each other during games and drills. On one occasion, this encouraging behavior led to Willie unexpectedly shouting *“Great job*, *Sallie”* and, on another occasion, to Sean suddenly giving Morris a high‐five and saying *“This is fantastic*, *Morris*. *You're really giving it all you've got on the pitch*. *I love it!”*.

The central role of the teacher also explains why we found high adherence to the “11 for Health” concept despite several schoolchildren not having any prior experience of football.

### 3.5 The importance of how praise partners are allocated

The last theme we identified, again relating to the microlevel of the ecological model, was the importance of how the praise partners within the “11 for Health” concept were allocated. We found that the teacher used the praise partner concept in a creative and neutral manner when pairing off the schoolchildren, eg, when she assigned praise partners based on children having the same color shoelaces, number of times doing leisure‐time activities per week or number of siblings. This creative and neutral approach defined the school children in a different way, regardless of football skills or school performance, and helped them regroup or find new social constellations. Consequently, praising became integrated within the class, as the children spontaneously began praising each other's skills more frequently. We found that this led to a supportive milieu, since every child had a praise partner keeping an eye on them and supporting their basic psychological needs of competence and relatedness.

## 4 DISCUSSION

This study aimed to gain a deeper understanding of the implementation facilitation of the “11 for Health” concept in a Danish 5^th^ grade school class. We provided an ecological description of the school environment, and examined and identified five factors influencing the implementation facilitation of the concept: (1) environmental and organizational factors; (2) familiarization with the concept for the teacher and schoolchildren; (3) previous clique formation; (4) the central role of the teacher; and (5) importance of how praise partners are allocated. This study contributes to the existing literature from a qualitative approach by expanding the knowledge about the “11 for Health” concept and contributes to the literature about active school concepts.

To summarize our results, we found that the autonomy‐supportive style of teaching and the way of applying conceptual elements, such as the praise partner concept, showed that the teacher played a key role in the implementation facilitation of “11 for Health”. The football clique of autonomously motivated boys also contributed to the implementation facilitation. Their familiarity with football was a resource, because their praising of classmates encouraged adherence. However, a general need for familiarization with games and drills at the beginning of the “11 for Health” concept and a differentiated focus on winning games and not always focusing on involvement, high rate of success and enjoyment on the part of the football clique was occasionally challenging. These challenges can be seen as a need for familiarization with the “11 for Health” concept and as a result of a competitive nature within the clique, as observed through their occasional negative use of football language, implicit behaviors, and actions. This language and these behaviors and actions were sometimes found to build barriers to other schoolchildren without football experience, possibly leading to a more controlled form of motivation, potentially jeopardizing the implementation facilitation of the “11 for Health” concept.

Within a Danish educational context with increasing weekly teaching hours,^35^ we found the teacher to be the key to the implementation facilitation of the “11 for Health” concept. A way to explain this was the teacher's ability to perform autonomy‐supportive teaching (AST) by creating an environment perceived as pleasant and intrinsically motivating and supportive of students’ basic psychological needs (BPN). We found this to have important pedagogical implications for schoolchildren's motivation,^36^ potentially leading to adaptive psychological outcomes^37^ and supporting them to eventually act as fully autonomous individuals. When familiarizing with the “11 for Health” concept, we found that the teacher was able to provide a rationale for the activities and encourage engagement. This way of teaching by offering hints, but not answers, to help schoolchildren overcome potential problems has also been found to be beneficial in other studies.^36^, ^38^ This might explain factors of the implementation facilitation of the “11 for Health” concept, as teachers focused on becoming more autonomy‐supportive are perceived as more autonomy‐supportive and significantly less controlling by their students.^39^ The schoolchildren were mostly found to support each other in improving, working together to learn new football skills, and trying their best.^40^ As a way of supporting these behaviors, we found the praise partner concept to be a valuable tool, again initiated by the teacher setting the stage for unconditional praising regardless of football skills and school performance. We found this to be a contributory factor in creating a supportive class milieu in which praising gradually became integrated, as every child had a praise partner supporting their basic psychological needs of competence and relatedness.^41^ These findings are supported elsewhere, underlining that teachers and peers are considered the most significant social agents in the school setting,^42^ while grounding PA interventions in self‐determination and basic psychological needs were found effective when developing and implementing school‐based interventions within Danish schools.^9^, ^43^

Another factor contributing to the implementation facilitation of the “11 for Health” concept was the previously formed clique of boys with an autonomous eagerness to organize and play football whenever possible. The boys’ autonomous motivation was characterized by a volitional engagement; a feeling that participation in the “11 for Health” concept was of their own choice and volition.^44^ However, we also found this to be a throughgoing communicative form, as most of the children used a specific verbal and bodily football language. Schein^18^ points out that culture—in this instance, the school, and the class environment—consists of layers of visibility. We found the football fellowship observable as artifacts and espoused values (norms and ideologies) that were communicated frequently, such as when the boys adjusted passes, shots, and tackles based on an awareness of whether other children were competent at football or not. This is important when focusing on necessary implementation and teaching factors, as over time norms and ideologies may contribute to a strong culture.^16^ However, meanings attached to the concept of enjoyment vary, and for some enjoyment means competing with peers of the same ability and also playing to win.^45^ It might therefore be expected that the clique of competitive boys was occasionally found to build barriers to schoolchildren outside the football fellowship, primarily through a negative use of football language. These somewhat taken‐for‐granted, underlying, and usually unconscious assumptions observed in the implicit behaviors and actions of the clique could potentially comprise the core of culture,^18^ as some children displayed more controlled forms of motivation. However, autonomous and controlled forms of motivation can coexist, meaning that schoolchildren might participate in “11 for Health” for a combination of reasons, some autonomous, others controlled.^46^ Where there is co‐existence, the teacher is again found to be the key person, as they control and support the autonomy within the “11 for Health” session, whereas classmates or peers to a greater extent support relatedness because they interact with their classmates throughout the day, not just during PE or classroom time.^37^

### 4.1 Methodological considerations

We selected the qualitative case‐study design to investigate a real‐life phenomenon within its real‐life context.^25^ We believe this approach was adequate for gaining a deeper understanding of the implementation facilitation of the “11 for Health” concept in a Danish 5^th^ grade school class. However, there is no precise consensus on the design and implementation of case studies, which made it crucial for us to create a dependable and defensible design.^47^ As an offshoot of earlier effect studies within the “11 for Health” concept, this study looked at the real‐time functioning of the environment. Observations of the participants and informal interviews complemented each other in providing an ecological description of the school environment. The two working models ensured an ecological description focusing exclusively on the school domain and teaching as inspired by Henriksen, Stambulova and Roessler.^17^

Two limitations of the study are worth mentioning. First, given the background of the authors, we acknowledge that depicting the environmental reality of the school environment was untenable, as we have to claim to be partially theory‐driven, making an objective assessment impossible.^48^ Second, the qualitative approach used in this study did not allow us to establish a rigorous causal relationship in terms of identifying factors influencing the environment and fully capturing all positive effects of the “11 for Health” concept. However, this case study can be seen as central to scientific development,^25^ as we have outlined important factors to be taken into account when considering the implementation facilitation of the “11 for Health” concept.

### 4.2 Perspectives

Football can be considered suitable within a school context because it is a well‐known activity and a widely practiced team sport. To strengthen this argument, “11 for Health” has been proven to provide effects on body composition, blood pressure, cardiovascular fitness, health knowledge, cognitive performance, well‐being, and enjoyment.^10^, ^11^, ^12^, ^13^, ^15^, ^49^ However, as we found the teacher to be a key factor in the implementation facilitation, we recommend that teachers apply AST when using the “11 for Health” concept. This means, among other things taking the schoolchild's perspective, vitalizing potential inner motivational resources; providing an explanatory rationale for the activities and drills; acknowledging and accepting negative effects during teaching; relying on and supporting informational and non‐pressuring language; and displaying patience when experiencing frustrated schoolchildren during teaching.^50^ Finally, we recommend that the concept should be endorsed at an organizational level, supporting the teacher in scheduling the “11 for Health” concept within the school curriculum.

## DISCLOSURE STATEMENT

No potential conflicts of interest were reported by the authors.
